# Percutaneous Endoscopic Gastrostomy Misplacement in the Transverse Colon of a Neurocognitively Compromised Patient

**DOI:** 10.7759/cureus.22063

**Published:** 2022-02-09

**Authors:** Robert Trenschel, Feargal Geraghty, Jacqueline Mirza, Daniel Chacon

**Affiliations:** 1 Medical School, Nova Southeastern University Dr. Kiran C. Patel College of Allopathic Medicine, Davie, USA; 2 Trauma, Kendall Regional Medical Center, Miami, USA; 3 School of Medicine, Ross University, Bridgetown, BRB

**Keywords:** neurocognitive dysfunction, general surgery complication, adult gastroenterology, surgery general, peg complication

## Abstract

Percutaneous endoscopic gastrostomy (PEG) tube placement is a widespread method of delivering sustained nutrition to individuals requiring long-term support. Multiple techniques exist to achieve this, and adverse events can arise if not done properly including but not limited to pneumoperitoneum and bowel perforation. Safeguard tactics exist to prevent these complications but they are not always successful. Herein, we explore a case of PEG tube misplacement through the transverse colon.

A 69-year-old male with a history of advanced dementia, cerebrovascular accident (CVA), and seizure disorder presented for a replacement of his malfunctioning PEG tube at a different site. On postoperative day one, the patient developed abdominal pain and shortness of breath. His subsequent imaging workup revealed pneumoperitoneum, and the patient ultimately underwent an exploratory laparotomy to repair the damage, washout his abdomen, and reinsert the PEG tube. Postoperatively, the patient had a lengthy hospital stay, which was complicated and prolonged by sepsis and mechanical ventilation.

The PEG tube placement procedure is not without its difficulties in all stages, pre- intra- and post-operatively, especially in patients with neurocognitive compromise, therefore, it is important to continue exploring methods to optimize the operation.

## Introduction

The most favorable method of feeding in those with functional gastrointestinal tracts who require long-term enteral support, typically greater than four weeks, is a percutaneous endoscopic gastrostomy (PEG) tube [1]. Major indications for PEG tube placement include cerebrovascular disease, dementia, and short bowel syndrome [2-3]. The PEG tube placement may be performed either in a surgical setting, by a gastroenterologist, or by a radiologist (percutaneous radiological gastrostomy - PRG). A number of techniques have been developed to ensure proper PEG placement, including the “pull,” “push,” and “introducer” (Russell) techniques, with the “pull” technique being the most utilized [3]. The absence of transillumination through the anterior abdominal wall raises concern for both correct and safe PEG placement, which, if the procedure is to continue, may result in serious complications including bowel perforation [1]. If transillumination fails, and a safe puncture site cannot be located, a PEG tube can be inserted through a process known as “safe tracking,” which includes the use of a fine 25-G guide needle that has been shown to reduce the potential for peri- and post-operative complications [4].

The timing and severity of complications associated with both PEG tube placement and utilization vary greatly. Examples of minor complications include the development of pneumoperitoneum or hernia, while examples of major complications include internal organ injury or bleeding [5]. Complications such as perforation occur with an incidence of <0.5%-1.8% [6]. Furthermore, the mortality rate of PEG insertion is <1% [7].

Here we discuss a case of PEG misplacement in the transverse colon leading to major complications and subsequently exploratory laparotomy, which resulted in both a prolonged course of mechanical ventilation and a prolonged hospital stay.

## Case presentation

**Figure 1:**
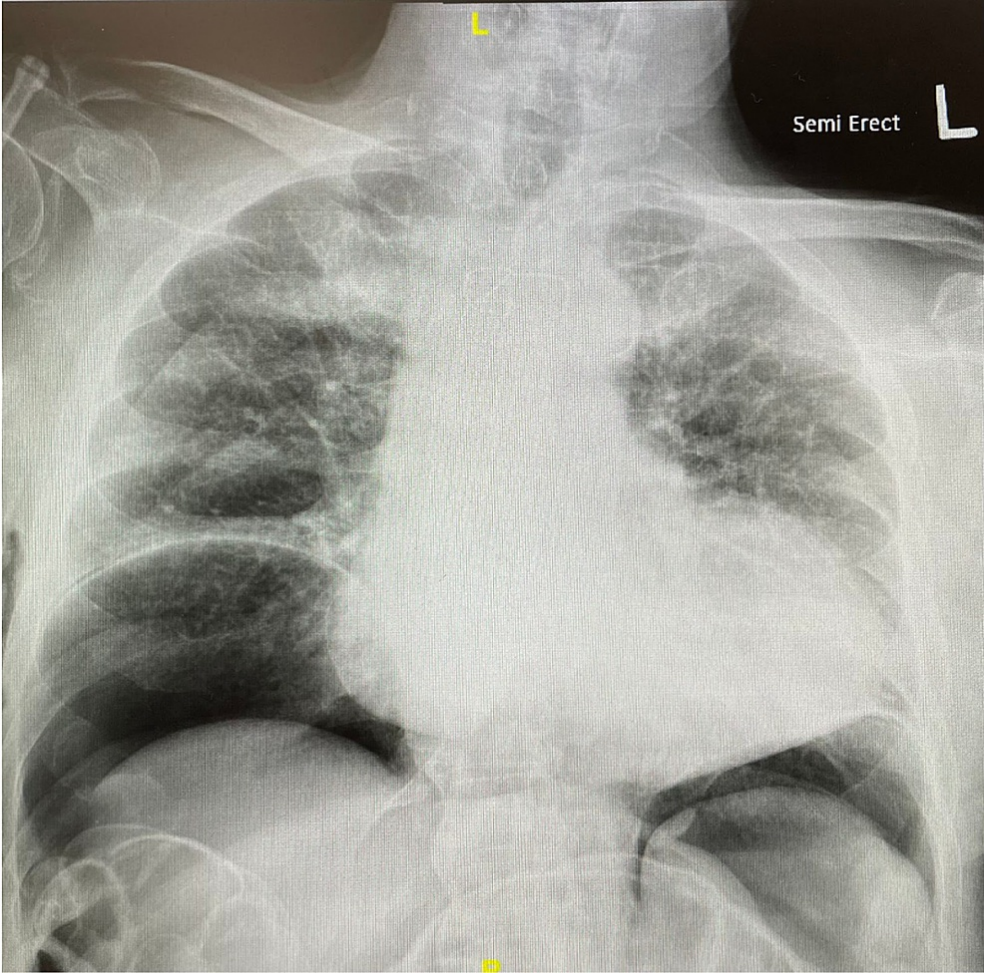
Admission CXR demonstrating pneumoperitoneum. CXR, chest X-ray

**Figure 2:**
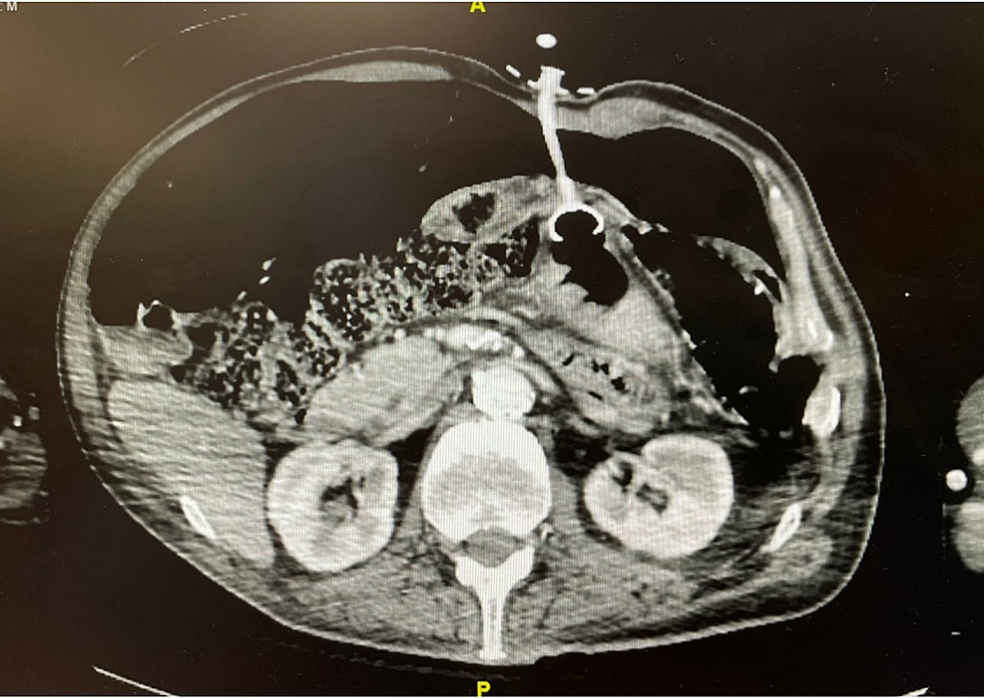
Admission CT abdomen and pelvis demonstrating misplaced PEG tube and pneumoperitoneum. PEG, percutaneous endoscopic gastrostomy

A 69-year-old male with a history of advanced dementia, cerebrovascular accident (CVA), and seizure disorder presented with a malfunctioning PEG tube that was placed five years prior to admission, in 2016. The patient had an endoscopic removal of that malfunctioning PEG tube, and it was subsequently replaced by gastroenterology (GI) at a different site. On postoperative day one following the new PEG placement, the patient endorsed abdominal pain and shortness of breath, however, the interaction was limited by the severity of his cognitive impairment. Work-up for the patient included labs, which revealed leukocytosis, but were otherwise grossly normal. Imaging for the patient included a chest X-ray, which revealed pneumoperitoneum. This finding prompted another consultation to GI and an abdominal and pelvic CT scan with both oral and IV contrast (Figures 1-2).

**Figure 3:**
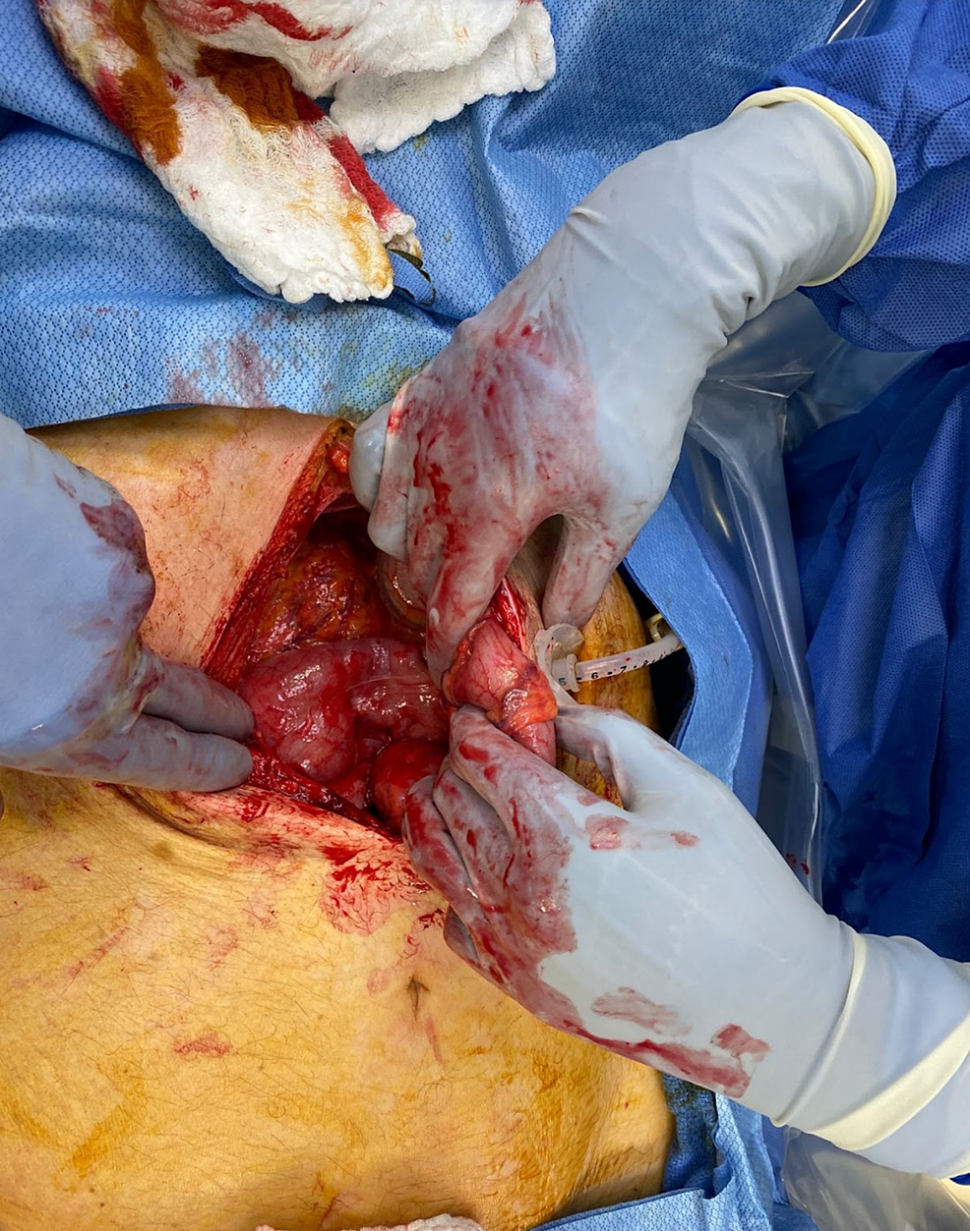
Exploratory laparotomy with intracolonic PEG tube. PEG, percutaneous endoscopic gastrostomy

The patient’s clinical picture was attributed to an intracolonic malposition of the PEG tube that had been placed the previous day. This finding prompted a general surgery consultation, and the team proceeded with an exploratory laparotomy. During the procedure, the patient’s PEG tube was replaced and repositioned. Intraoperatively, the patient received an abdominal washout, repair of the anterior and posterior transverse colon perforation, repair of the anterior stomach, repair of the mesentery, and the insertion of a 20 French open gastrostomy tube in the anterior stomach (Figure 3). No surgical complications were encountered during the procedure.

**Figure 4:**
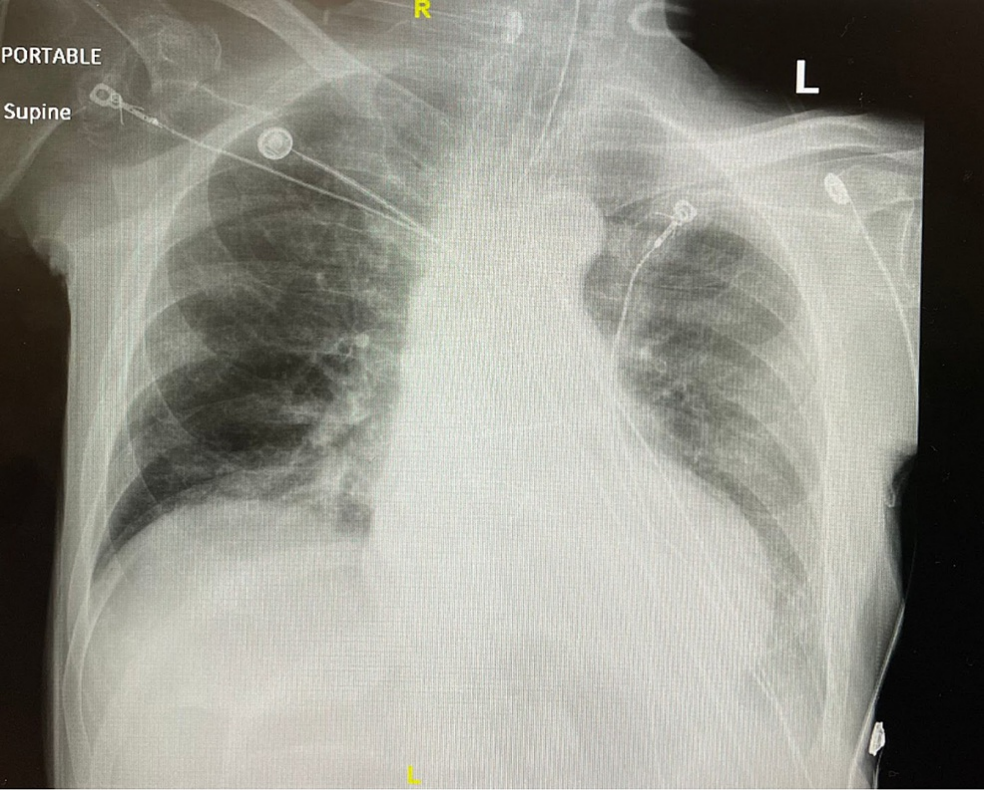
Day five postoperative CXR with resolved pneumoperitoneum. CXR, chest X-ray

The patient’s postoperative course was complicated by sepsis, and acute respiratory failure necessitating mechanical ventilation to stabilize the airway. Five days postoperatively, the patient had a normal upper GI series. Additionally, the patient’s postoperative chest X-ray revealed no abnormalities with a resolution of the pneumoperitoneum (Figure 4). The surgical intervention was considered appropriate, no further interventions with the PEG were required, and the patient tolerated normal tube feeds.

## Discussion

The advent of PEG tube feeding in the late 20th century has provided a revolutionary new method of providing enteral nutrition to patients with the inability to swallow, with the first procedure being reported by Gauderer in 1980 [8]. The procedure has a remarkable success rate, which reaches 95%. Even so, complications are not uncommon, with a procedure-related morbidity rate of 9.4% and a mortality rate of 0.53% [9]. 

The PEG tube placement is not without difficulties both pre-and postoperatively; therefore, it is important to explore ways to make the procedure more effective at increasing patients’ wellbeing. Major risk factors present an obstacle for safe and efficacious PEG tube placement. Mortality rates following PEG tube placement have been identified in the literature, and are largely associated with advancing age, high Charlson comorbidity score, low serum albumin, and high level of cognitive impairment [10]. Individuals with advanced dementia and inadequate oral intake represent a special population, PEG placement for whom warrants a conversation between the caregivers and the providers. At present, there is a paucity of evidence that suggests any long-term benefit of tube feeding amongst patients with advanced cognitive decline [2, 11]. In fact, the European Society of Gastrointestinal Endoscopy (ESGE) recommends against PEG tube placement in patients with advanced dementia; therefore, patients like the one presented in this case should have robust cognitive assessment done prior to PEG placement, or replacement [12]. 

Intraoperatively, it is imperative to take precautions and to do rigorous postoperative monitoring of patients to ensure proper placement of their PEG tube. The American College of Gastroenterology does not specifically recommend esophageal physiologic testing as part of PEG tube placement follow up; rather, this modality of testing is more commonly used to evaluate obstructive symptoms, like dysphagia and regurgitation, gastroesophageal reflux disease (GERD) symptoms, and behavioral symptoms [13]. Pneumoperitoneum is common in the first few postoperative days, occurring in as many as 50% of cases. According to the European Society of Clinical Nutrition and Metabolism, even in the presence of abdominal pain, the recommendation is conservative management without surgical exploration [14]. 

Misplacement is an under-reported complication of PEG tube insertion, with common sites of injury being the transverse colon, small bowel, and liver. The liver is one of the rarer locations for malpositioning, with very few cases having been reported in the literature, and being relatively difficult to diagnose [15]. Colonic perforation, however, is slightly more common. A literature search of cases reporting colonic perforation secondary to PEG tube malpositioning yielded one case reporting intractable diarrhea as a result of the misplacement; the placement of which only being localized after colonoscopy [16]. Cases have been described where a malpositioned PEG tube in the transverse colon, visualized via colonoscopy, have been treated endoscopically due to extensive contraindications to surgery [17]. Cases like these offer a window of opportunity not only for patients who are not surgical candidates but also for those for whom surgery presents an increased risk of morbidity and mortality. Further reporting of cases like these will add greatly to the existing literature and help shape postoperative questioning and management. 

## Conclusions

The PEG tube placement is a surgical procedure that is not without complications. Although commonly done, steps including reviewing preoperative imaging and safe track placement are key. Here we present a neurocognitively compromised patient who arrived at our center with complications surrounding PEG tube misplacement in the transverse colon. Data lacking clear indications and contraindications to PEG placement present a challenge for patient management. This is especially highlighted by the presented case, given that the patient’s cognitive status likely complicated the decision to initiate enteral nutrition, the decision of which ultimately put him at risk for developing potentially life-threatening complications, including misplacement. This case represents a call to action for healthcare professionals to add to the growing body of research surrounding both PEG placement and misplacement, which will only become more essential as the population continues to age. 

## References

[1] Baile-Maxía S, Medina-Prado L, Bozhychko M (2020). Endoscopic ultrasound-guided percutaneous endoscopic gastrostomy. Dig Endosc.

[2] Goldberg LS, Altman KW (2014). The role of gastrostomy tube placement in advanced dementia with dysphagia: a critical review. Clin Interv Aging.

[3] Rahnemai-Azar AA, Rahnemaiazar AA, Naghshizadian R, Kurtz A, Farkas DT (2014). Percutaneous endoscopic gastrostomy: indications, technique, complications and management. World J Gastroenterol.

[4] Chang WK, Hsieh TY (2013). Safety of percutaneous endoscopic gastrostomy in high-risk patients. J Gastroenterol Hepatol.

[5] Hucl T, Spicak J (2016). Complications of percutaneous endoscopic gastrostomy. Best Pract Res Clin Gastroenterol.

[6] Gupta N, Likhtshteyn M, Kancharla P, Iqbal S, Al-Ani F, Grossman E (2019). 2754 A gaping hole in the stomach - a rare PEG tube complication. Am J Gastroenterol.

[7] Guloglu R, Taviloglu K, Alimoglu O (2003). Colon injury following percutaneous endoscopic gastrostomy tube insertion. J Laparoendosc Adv Surg Tech A.

[8] Gauderer MW, Ponsky JL, Izant RJ Jr (1980). Gastrostomy without laparotomy: a percutaneous endoscopic technique. J Pediatr Surg.

[9] Potack JZ, Chokhavatia S (2008). Complications of and controversies associated with percutaneous endoscopic gastrostomy: report of a case and literature review. Medscape J Med.

[10] Cowen ME, Simpson SL, Vettese TE (1997). Survival estimates for patients with abnormal swallowing studies. J Gen Intern Med.

[11] Sampson EL, Candy B, Jones L (2009). Enteral tube feeding for older people with advanced dementia. Cochrane Database Syst Rev.

[12] Arvanitakis M, Gkolfakis P, Despott EJ (2021). Endoscopic management of enteral tubes in adult patients - part 1: definitions and indications. European Society of Gastrointestinal Endoscopy (ESGE) Guideline. Endoscopy.

[13] Gyawali CP, Carlson DA, Chen JW, Patel A, Wong RJ, Yadlapati RH (2020). ACG clinical guidelines: clinical use of esophageal physiologic testing. Am J Gastroenterol.

[14] Löser C, Aschl G, Hébuterne X (2005). ESPEN guidelines on artificial enteral nutrition--percutaneous endoscopic gastrostomy (PEG). Clin Nutr.

[15] Chhaparia A, Hammami MB, Bassuner J, Hachem C (2018). Trans-hepatic percutaneous endoscopic gastrostomy tube placement: a case report of a rare complication and literature review. Gastroenterol Res.

[16] Burke DT, Geller AI, Carayannopoulos AG, Goldstein R (2011). Inadvertent percutaneous endoscopic gastrostomy tube placement through the transverse colon to the stomach causing intractable diarrhea: a case report. Diagn Ther Endosc.

[17] Campbell JP, Klein D, Gupta V (2019). 1862 novel endoscopic repair of a percutaneous endoscopic gastrostomy tube malpositioned through the transverse colon. Am J Gastroenterol.

